# Evaluation of genetic gains of some quantitative characters in Egyptian cotton cross (Giza 86 × Menoufi) under water deficit stress

**DOI:** 10.1038/s41598-022-18966-3

**Published:** 2022-09-08

**Authors:** Mohamed S. Abd EL-Aty, Mohamed A. Al-Ameer, Mohamed M. Kamara, Mohamed M. Elmoghazy, Omar M. Ibrahim, Ammar AL-Farga, Amira M. El-Tahan

**Affiliations:** 1 Agronomy Department, Faculty of Agriculture, Kafr El-Sheikh University, P.O. 33516, Kafr El-Shiekh, Egypt; 2 Cotton Breeding Department, Cotton Research Institute, Agriculture Research Center, Giza, Egypt; 3 Plant Production Department, Arid Lands Cultivation Research Institute, The City of Scientific Research and Technological Applications, SRTA-City, Borg El Arab, Alexandria Egypt; 4 Department of Biochemistry, College of Science, University of Jeddah, Jeddah, Saudi Arabia

**Keywords:** Agricultural genetics, Plant breeding

## Abstract

This work was carried out to select cotton genotypes adapted to semi-arid climate conditions cultivated under irrigation for high yields and the standards of the fiber quality properties required by the textile industry. Also to determine the predicted and realized gains from different selection indices to improve some economic characters under water stress conditions. Except for lint percentage and Pressley index, F4 generation reduced PCV and GCV values for all studied characters due to reduction in genetic variability and heterozygosity due to different selection procedures that exhausted a significant part of variability. Except for fiber length and micronaire reading, mean performance in the F4 generation was revealed to be higher than those in the F3 generation for all studied characters. However, micronaire reading was lower (desirable) in F4 than F3 generation. Generally, genotypic correlations were higher than phenotypic correlations. Direct selection for lint index (Ped.3) was the most efficient in improving lint cotton yield/plant and bolls/plant. However, the multiplicative index involving all studied characters (I.5) exhibited the highest values for boll weight. Also, the Ped.2 index (direct selection for lint percentage) proved to be the most efficient in improving seed and lint indexes. Direct selection for lint cotton yield/plant (Ped.1) could produce the highest desirable values for lint percentage and seed per boll with a relatively reasonable yield. A selection index involving yield and its components (I.3) is recommended in improving uniformity index, fiber strength, and micronaire reading. The superior five families released from these indices in F4 generation exceeded the better parent for lint cotton yield/plant, bolls/plant, boll weight, seeds/boll, lint index, and reasonable fiber traits. These families could be continued to further generations as breeding material for developing water deficit tolerant genotypes.

## Introduction

"White gold" is the famous name of cotton; it takes this fame from its importance in the economy. Cotton is not only the essential fiber crop in the world but the second most crucial oilseed. Also, cotton is a fiber and oil crop grown in more than 70 countries worldwide, which plays an essential role in the global economy. India is the largest producer, with an output of 5.9 million tons, followed by China, the USA, Pakistan, and Brazil^1^.

Cotton is the main cash crop of Egypt. Since 1820, there has been no crop than cotton, continuing in the Egyptian people's life and survival. It has an essential role in the national economy in contributing to trade, textile industry, employment, and foreign exchange. Increasing productivity per unit area is the requirement for the sustenance of domestic and export needs. Expression of various economic traits often varies with the breeding materials used and environmental conditions^2–4^.

In Egypt, water scarcity, especially at the ends of the canals, is a significant factor limiting the cultivation of the cotton crop^2,5^. Drought is common abiotic stress during the cotton growing season, which causes a series of adverse effects on cotton growth, yield, and fiber quality^2,6,7^. Cotton is a dreadfully drought-sensitive crop causing incentive reduction in yield because drought stress is a complex phenomenon that affects the physiology of the cotton plant^6^. Also, cotton is a very susceptible plant to the quantity of irrigation water, and therefore, irrigation management is very complicated. The flowering and boll-forming stage is the cotton plants' critical yield determinant period. Water stress occurring during this stage will undoubtedly seriously affect cotton development and final productivity^8–10^. Water-deficit stress affects physiological processes in plants, resulting in alterations in photosynthetic rate, transpiration rate, stomatal conductance, carboxylation efficiency, and water use efficiency in plants**.** Most practical drought tolerance breeding programs emphasize direct selection for yield under stress. However, underwater stress, high-yielding genotypes could likely be low-yielding under well-watered environments^11–13^**.** Also, ^14–16^observed that genotypes bred in optimum conditions are not likely to sustain yield genotype-environment interaction, and selection only under water stress conditions is fruitful.

Direct selection based on yield only is mainly difficult practiced in cotton breeding, so yield is a complex trait and highly affected by environmental conditions; however, the presence of genotype x environment interactions reduces the efficiency of using yield as the sole selection criterion and thus, complicates the efforts of selection^17–19^**.** In addition to the environmental effects, other factors such as polygenic nature, low heritability, linkage, and non-additive gene action may make the selection less efficient, mainly in early segregation generations^20–22^. The selection index technique was proposed by^23,24^ to be used in the simultaneous improvement of several traits and to select for relatively more heritable correlated characteristics. Furthermore, the selection index aims to determine the most valuable genotypes and the most suitable combination of traits plants^8,12,25,26^.

Some comparisons of the indices with direct selection conclude that using indices as a selection criterion achieves superior results. Several authors confirmed the efficacy of the selection index among them^11,14,16,27–31^. It could be concluded that the selection index method was more efficient in isolating the superior elite families in most studies traits. We can depend on this method from selection in scientific programmers to obtain elite genotypes superior yield and fiber traits together. On the other hand, the pedigree selection method was inferior to detecting the superior genotypes^17–22^.

The first objective of the research reported herein was to select cotton genotypes adapted to semi-arid climate conditions cultivated under irrigation for high yields and the standards of the fiber quality properties required by the textile industry. The second objective of the research was to determine the predicted and realized gains from different selection indices to improve some economic characters under water stress conditions.

## Results

Heritability values in broad-sense, phenotypic (PCV), and genotypic (GCV) coefficients of variation and mean performance for all the studied traits are presented in Table 1. Heritability was high, over 50% for all the studied features in F_2,_ F_3,_ and F_4_ generations expect BW, S/B, and SI in the F_2_ generation. B/P, SI, LI, UI, FL, and PI showed a decrease in heritability in a broad sense from F_3_ to F_4_ generations. On the other hand, LCY/P, BW, S/B, LP%, and MR increased heritability values from F_3_ to F_4_ generations. The observed phenotypic and genotypic coefficients of variation were more significant in F_2_ and F_3_ than in F_4_ for all the studied traits. Except for LP% and PI, it is interesting to mention that F_4_ generation reduced PCV and GCV values for all studied characters.

**Table 1:** Estimates of broad-sense heritability (h^2^_b_), phenotypic (PCV), and genotypic (GCV) coefficients of variation, means, and standard errors (${\text{S}}\overline{x}$) for the eleven studied characters in F_2_, F_3,_ and F_4_ generations.

Character	Generation	Mean ± SE	PCV%	GCV%	h^2^_b_
LY/P (g)	F_2_	19.55 ± 0.43	44.26	34.67	60.84
	F_3_	11.79 ± 1.55	31.76	28.91	82.85
	F_4_	16.65 ± 0.49	8.93	8.41	89.11
B/P	F_2_	16.41 ± 0.33	41.41	30.20	52.74
	F_3_	11.29 ± 1.34	29.94	27.49	84.31
	F_4_	14.77 ± 0.41	6.02	5.33	78.14
BW (g)	F_2_	3.17 ± 0.02	11.21	6.72	38.46
	F_3_	3.14 ± 0.13	6.68	5.32	63.54
	F_4_	3.33 ± 0.08	4.14	3.43	69.63
S/B	F_2_	21.03 ± 0.13	12.86	6.43	30.78
	F_3_	19.03 ± 0.85	6.55	4.77	53.04
	F_4_	19.32 ± 0.39	4.08	3.55	75.39
SI (g)	F_2_	9.57 ± 0.04	9.21	5.72	39.74
	F_3_	10.54 ± 0.19	8.88	8.69	95.96
	F_4_	11.01 ± 0.18	5.14	4.89	90.59
LP%	F_2_	37.30 ± 0.10	5.36	2.64	86.50
	F_3_	36.44 ± 0.18	3.12	2.69	74.03
	F_4_	36.62 ± 0.55	3.40	3.05	80.39
LI (g)	F_2_	3.95 ± 0.02	10.81	3.99	50.00
	F_3_	6.05 ± 0.74	9.56	7.85	80.45
	F_4_	6.37 ± 0.20	6.28	5.50	74.68
UI%	F_3_	84.15 ± 0.45	1.22	1.10	80.91
	F_4_	84.42 ± 0.31	0.83	0.74	80.14
FL (mm)	F_3_	33.52 ± 0.45	3.58	3.31	85.59
	F_4_	32.27 ± 0.44	2.74	2.39	76.20
PI	F_3_	9.54 ± 0.12	3.16	2.91	84.64
	F_4_	10.91 ± 0.23	3.55	2.84	63.69
MR	F_3_	4.16 ± 0.45	4.99	4.43	78.33
	F_4_	4.11 ± 0.04	3.76	3.57	90.54

Mean performances for the studied traits in F_2_, F_3,_ and F_4_ generations are presented in Table 1. Except FL and MR, mean performance in F_4_ generation revealed higher than in F_3_ generation for all studied characters. However, MR was lower (desirable) in F_4_ than F_3_ generation.

### Phenotypic and genotypic correlation coefficients

The coefficient of phenotypic and genotypic correlations among different character combinations are given in Table 2. The results revealed that B/P, BW, S/B, and LP% had positive and significant with lint cotton yield/plant at most studied generations. Also, fiber length was positively correlated with LCY/P in F_3_ and F_4_ generations. B/P was negatively associated with SI and LI in both r_p_ and r_g_. BW positively and significantly correlated with SI, LI, and MR in r_p_ and r_g_. Phenotypic and genotypic correlation coefficients for S/B with LP% were positive and increased from F_2_ to F_4_ generations, but S/B showed negative associations with SI and LI. SI exhibited a positive relationship with LI but showed negative associations with LP%. Except for the negative relationship between PI and MR in F_3_, there is no clear relationship between the remaining fiber traits and each other.

**Table 2:** Phenotypic(r_p_) (above diagonal) and Genotypic(r_g_) (below diagonal) correlation coefficients in F_2_, F_3,_ and F_4_ generations between all pairs of studied traits.

Character	Generation	LY/P (g)	B/P	BW (g)	S/B	SI (g)	LP%	LI (g)	UI%	FL (mm)	PI	MR
LY /P (g)	F_2_		0.943**	0.310**	0.282**	− 0.023	0.215**	0.151**	–	–	–	–
	F_3_		0.969**	0.268**	0.453**	− 0.167*	0.195**	− 0.072	− 0.062	0.046	− 0.075	0.132*
	F_4_		0.657**	0.388**	0.414**	− 0.125	0.543**	0.350**	0.149	0.504**	− 0.314*	− 0.015
B/P	F_2_	0.936**		0.065	0.091	− 0.06	0.070	0.004	–	–	–	–
	F_3_	0.975**		0.063	0.418**	− 0.268**	0.099	− 0.240**	− 0.066	0.001	− 0.006	0.051
	F_4_	0.662**		− 0.137	0.187	− 0.170	− 0.025	− 0.196	0.318*	0.550**	0.192	− 0.005
BW (g)	F_2_	0.331**	0.038		0.676**	0.218**	0.099*	0.261**	–	–	–	–
	F_3_	0.205	0.015		0.185**	0.606**	− 0.060	0.610**	0.142*	0.239**	− 0.219**	0.296**
	F_4_	0.476*	− 0.252		0.085	0.402**	0.270*	0.532**	− 0.083	0.195	− 0.348**	0.324*
S/B	F_2_	0.396**	0.249**	0.393**		− 0.433**	0.036	− 0.339**	–	–	–	–
	F_3_	0.507**	0.545**	− 0.311**		− 0.632**	0.211**	− 0.548**	− 0.203**	− 0.159*	− 0.093	0.070
	F_4_	0.520*	0.230	0.010		− 0.804**	0.409**	− 0.313*	0.056	0.245*	− 0.057	− 0.031
SI (g)	F_2_	− 0.240**	− 0.316**	0.297**	− 0.897**		− 0.260**	0.642**	–	–	–	–
	F_3_	− 0.184	− 0.299**	0.787**	− 0.803**		− 0.411**	0.811**	0.274**	0.277**	− 0.011	0.096
	F_4_	− 0.138	− 0.204	0.465*	− 0.957**		− 0.350**	0.489**	− 0.144	− 0.172	0.009	0.343**
LP%	F_2_	0.284**	0.085	0.221**	0.189**	− 0.354**		0.642**	–	–	–	–
	F_3_	0.225*	0.136	− 0.12	0.419**	− 0.489**		0.162*	− 0.093	− 0.014	− 0.295**	0.215**
	F_4_	0.625**	− 0.117	0.263	0.524*	− 0.408*		0.638**	− 0.010	0.118	− 0.717**	− 0.272*
LI (g)	F_2_	0.117*	− 0.151**	0.483**	− 0.441**	0.331**	0.768**		–	–	–	–
	F_3_	− 0.094	− 0.290*	0.871**	− 0.656**	0.869**	− 0.007		0.211**	0.257**	− 0.213**	0.250**
	F_4_	0.420*	− 0.343	0.593*	− 0.419*	0.505*	0.574*		− 0.105	− 0.019	− 0.617**	− 0.043
UI%	F_3_	− 0.076	− 0.085	0.196	− 0.304**	0.308**	− 0.127	0.268*		0.582**	0.053	0
	F_4_	0.118	0.323	− 0.075	0.087	− 0.178	− 0.057	− 0.190		0.531**	0.142	− 0.087
FL	F_3_	0.071	0.015	0.336**	− 0.183	0.291*	− 0.057	0.268*	0.590**		0.123	− 0.122
	F_4_	0.528*	0.605**	0.356	0.425*	− 0.195	0.131	− 0.031	0.449*		0.247*	− 0.026
PI	F_3_	− 0.084	− 0.001	− 0.295**	− 0.14	− 0.01	− 0.372**	− 0.252*	0.067	0.128		− 0.909**
	F_4_	− 0.446*	0.255	− 0.602**	0.126	0.014	− 1.002**	− 0.883**	0.307	0.316		0.016
MR	F_3_	0.148	0.046	0.411**	0.122	0.101	0.282*	0.300**	0.005	− 0.155	− 0.927**	
	F_4_	− 0.013	0.011	0.447*	− 0.093	0.349	− 0.273	− 0.038	− 0.089	0.019	0.189	

* and ** mean significant at 0.05 and 0.01 levels of probability, respectively.

### Predicted and realized gains from the selection

Predicted and realized advances from selection procedures for yield and yield components are presented in Table 3. The highest predicted genetic advances in F_2_, F_3,_ and F_4_ generations for LCY/P and B/P were obtained with Ped._1_ and I._1_. The high genetic correlation (more than 0.93) between LCY/P and B/P Table 2 could explain the improvement of both LCY/P and B/P using direct selection for lint cotton yield/plant (Ped._1_) and selection index involving yield and its components (I._1_). The highest realized and predicted genetic advances in the F_4_ generation were obtained with the index Ped._1_(direct selection for LCY/P). The results from Ped._1_ had already been expected, since the positive correlations between lint index with LCY/P and B/P in F_4_, these results indicate the genetic variation for lint cotton yield/plant in early generations didn’t exhaust enough, and the improvement of yield/plant could be continued in further generations.

**Table 3:** Predicted and realized gains from the different selection procedures for yield and yield components in the three segregating generations.

Selection procedures	Predicted gain F_2_			Predicted gain F_3_			Realized gain F_4_		Predicted gain F_4_		
	X_S_	G_S_	G_S_%	X_S_	G_S_	G_S_%	G_S_	G_S_%	X_S_	G_S_	G_S_%
**Lint cotton yield/plant (g)**^**a**^											
Ped._1_	40.84	12.95	66.24	18.99	5.96	50.59	1.85	11.08	18.50	1.64	9.88
Ped._2_	23.97	2.69	13.73	12.26	0.39	3.32	0.85	5.11	17.50	0.76	4.55
Ped._3_	21.71	1.31	6.72	13.81	1.67	14.19	0.78	4.66	17.43	0.69	4.16
I._1_	39.05	11.86	60.66	18.99	5.96	50.59	1.66	9.94	18.31	1.48	8.86
I._2_	–	–	–	12.64	0.71	5.99	0.37	2.21	17.02	0.33	1.97
I._3_	–	–	–	13.40	1.33	11.31	1.06	6.36	17.71	0.94	5.67
I._4_	–	–	–	18.34	5.43	46.05	1.54	9.22	18.19	1.37	8.22
I._5_	–	–	–	18.88	5.87	49.82	1.64	9.84	18.29	1.46	8.77
**Bolls/plant**^**b**^											
Ped._1_	31.13	7.76	47.21	16.96	4.78	42.29	0.90	6.07	15.67	0.70	4.73
Ped._2_	17.53	0.58	3.55	11	− 0.25	− 2.19	0.03	0.20	14.80	0.02	0.15
Ped._3_	15.53	− 0.47	− 2.87	12.33	0.88	7.76	0.10	0.65	14.87	0.07	0.50
I._1_	26.8	5.47	33.3	16.96	4.78	42.29	0.63	4.27	15.40	0.49	3.32
I._2_	–	–	–	11.5	0.17	1.54	0.63	4.27	15.40	0.49	3.32
I._3_	–	–	–	12.25	0.81	7.14	0.43	2.91	15.20	0.33	2.26
I._4_	–	–	–	16.71	4.56	40.42	0.70	4.72	15.47	0.54	3.68
I._5_	–	–	–	16.79	4.64	41.04	0.70	4.72	15.47	0.54	3.68
**Boll weight (g)**^**c**^											
Ped._1_	3.36	0.07	2.26	3.36	0.14	4.41	0.045	1.36	3.38	0.03	1.03
Ped._2_	3.26	0.03	1.09	3.15	0.01	0.25	− 0.001	− 0.02	3.33	0.00	0.07
Ped._3_	3.42	0.09	2.97	3.34	0.12	3.96	0.065	1.96	3.40	0.05	1.45
I._1_	3.5	0.13	3.96	3.36	0.14	4.41	0.007	0.22	3.34	0.01	0.23
I._2_	–	–	–	3.34	0.12	3.94	0.007	0.20	3.34	0.01	0.22
I._3_	–	–	–	3.23	0.06	1.83	0.041	1.24	3.37	0.03	0.95
I._4_	–	–	–	3.3	0.1	3.2	0.040	1.20	3.37	0.03	0.92
I._5_	–	–	–	3.36	0.14	4.46	0.102	3.06	3.43	0.07	2.22
**Seeds/boll**^**d**^											
Ped._1_	22.6	0.49	2.32	19.63	0.32	1.66	0.21	1.10	19.53	0.16	0.85
Ped._2_	20.6	− 0.13	− 0.6	19.33	0.16	0.84	0.35	1.79	19.67	0.26	1.37
Ped._3_	19.33	− 0.52	− 2.45	17.79	− 0.66	− 3.45	− 0.59	− 3.04	18.73	− 0.44	− 2.27
I._1_	23	0.61	2.91	19.63	0.32	1.66	0.28	1.45	19.60	0.21	1.11
I._2_	–	–	–	19.04	0.01	0.03	0.15	0.76	19.47	0.11	0.59
I._3_	–	–	–	18.58	− 0.24	− 1.25	0.35	1.79	19.67	0.26	1.37
I._4_	–	–	–	19.46	0.23	1.19	0.35	1.79	19.67	0.26	1.37
I._5_	–	–	–	19.63	0.32	1.66	− 0.05	− 0.28	19.27	− 0.04	− 0.19
**Seed index (g)**^**e**^											
Ped._1_	9.57	0.002	0.03	10.89	0.33	3.14	− 0.05	− 0.42	10.96	− 0.04	− 0.41
Ped._2_	9.3	− 0.11	− 1.12	10.06	− 0.46	− 4.37	− 0.39	− 3.52	10.62	− 0.35	− 3.21
Ped._3_	10.79	0.49	5.17	11.84	1.25	11.81	0.48	4.32	11.49	0.43	3.89
I._1_	9.68	0.04	0.46	10.89	0.33	3.14	− 0.14	− 1.28	10.87	− 0.13	− 1.19
I._2_	–	–	–	11.23	0.66	6.23	0.03	0.28	11.04	0.03	0.23
I._3_	–	–	–	11.00	0.43	4.11	− 0.29	− 2.68	10.72	− 0.27	− 2.45
I._4_	–	–	–	10.79	0.23	2.2	− 0.18	− 1.67	10.83	− 0.17	− 1.53
I._5_	–	–	–	10.88	0.32	3.06	0.25	2.23	11.26	0.22	1.99
**Lint index (g)**^**f**^											
Ped._1_	38.2	0.78	2.08	36.82	0.28	0.77	0.59	1.60	37.21	0.47	1.29
Ped._2_	41.46	3.6	9.64	38.41	1.45	3.98	1.50	4.09	38.12	1.20	3.29
Ped._3_	40.01	2.34	6.29	36.89	0.33	0.91	0.89	2.44	37.51	0.72	1.96
I._1_	39.83	2.19	5.87	36.82	0.28	0.77	0.76	2.08	37.38	0.61	1.67
I._2_	–	–	–	36.28	− 0.12	− 0.33	− 0.62	− 1.68	36.00	− 0.50	− 1.35
I._3_	–	–	–	37.06	0.46	1.26	1.04	2.85	37.66	0.84	2.29
I._4_	–	–	–	36.86	0.31	0.85	0.49	1.33	37.11	0.39	1.07
I._5_	–	–	–	37.04	0.44	1.2	0.49	1.33	37.11	0.39	1.06
**Lint index (g)**^**g**^											
Ped._1_	5.93	0.12	2.04	6.34	0.23	3.83	0.13	1.98	6.50	0.09	1.43
Ped._2_	6.59	0.44	7.69	6.37	0.25	4.2	0.22	3.51	6.59	0.16	2.57
Ped._3_	7.17	0.72	12.69	6.99	0.76	12.54	0.57	8.92	6.94	0.42	6.61
I._1_	6.4	0.34	6.04	6.34	0.23	3.83	0.12	1.86	6.49	0.09	1.34
I._2_	–	–	–	6.34	0.24	3.9	− 0.15	− 2.37	6.22	− 0.12	− 1.81
I._3_	–	–	–	6.46	0.33	5.52	0.15	2.40	6.52	0.11	1.74
I._4_	–	–	–	6.29	0.19	3.18	0.02	0.28	6.39	0.01	0.16
I._5_	–	–	–	6.4	0.28	4.61	0.27	4.31	6.64	0.20	3.17

^a^The simple correlation between predicted and realized gains = 0.847**.

^b^The simple correlation between predicted and realized gains = 0.769*.

^c^The simple correlation between predicted and realized gains = 0.434 ns.

^d^The simple correlation between predicted and realized gains = 0.667 ns.

^e^The simple correlation between predicted and realized gains = − 0.800*.

^f^The simple correlation between predicted and realized gains = 0.813*.

^g^The simple correlation between predicted and realized gains = 0.820*.

On the other hand, the lowest realized and predicted genetic advances in F_4_ generation for LCY/P was obtained with the index I_.2_ (the index involving BW, S/B, SI, fiber length, and PI). There was disagreement between the eight indexes regarding the predicted and realized responses (simple correlation between predicted and realized gains = 0.874 for LCY/P and = 0.769 for B/P). also the predicted improvements in F_3_ were higher than the realized gains in F_4_, which indicate the predominance of additive and dominance genetic variances in the inheritance for LCY/P and B/P.

Regarding BW and S/B Table 3, the highest predicted genetic advances in F_2_ and F_3_ generations were obtained with the index I._1_. Also, I._4_ for S/B and I._5_for BW gave the highest realized and predicted genetic advances in the F_4_ generation. The simple correlation between predicted (F_3_) and realized (F_4_) gains was 0.434 for BW and 0.667 for S/B at the eight indexes; also, the predicted increases in F_3_ were higher than the realized gains in F_4_ at most selection procedures.

For SI and LI Table 3, a complete agreement was found for these two traits, as Ped._3_ (direct selection for LI) gave the highest expected genetic gains in F_2_, F_3,_ and F_4_ generations_;_ this is due to the strong genetic correlation between both seed index and lint percentage with lint index. In contrast, Ped._2_ (direct selection for LP% showed the lowest realized and predicted genetic advances in all generations for SI and LI. The predicted gains in F_3_ were higher than the realized gains in F_4_ at most selection procedures. The simple correlation between predicted and realized gains was 0.820 at the eight indexes for LI. Thus, there was relatively agreement between this trait's predicted and realized responses.

Considering LP% Table 3, maximum predicted advance was obtained by using Ped._2_ (direct selection for LP%) in F_2_, F_3,_ and F_4_ generations. Thus, direct selection for LP% is recommended in itself improvement. The simple correlation between predicted and realized gains was 0.813 at the eight indexes. There was a fluctuation in the differences between predicted and actual genetic increases in value and direction.

Regarding UI and fiber length Table 4, the selection procedure involved LP%, LI, FL, and UI (I._3_) gave the highest predicted gains in the F_3_ generation. Also, I._2_ (the index involving BW, S/B, SI, FL, and PI) showed the high realized and predicted genetic advances in the F_4_ generation. The eight indexes exhibited differences between predicted and actual genetic gains in both UI and fiber length value and direction. The simple correlation between predicted and realized gains was 0.680 for UI and 0.702 for fiber length.

**Table 4:** Predicted and realized gains from the different selection procedures for fiber properties in the three segregating generations.

Selection procedures	Uniformity index							
	Predicted gain F_3_			Realized gain F_4_		Predicted gain F_4_		
	X_S_	G_S_	G_S_%	G_S_	G_S_%	X_S_	G_S_	G_S_%
Ped._1_	83.92	− 0.19	− 0.22	0.14	0.17	84.56	0.11	0.13
Ped._2_	84.35	0.16	0.19	− 0.06	− 0.07	84.36	− 0.05	− 0.06
Ped._3_	84.93	0.62	0.74	0.01	0.01	84.43	0.01	0.01
I._1_	83.92	− 0.19	− 0.22	− 0.29	− 0.34	84.13	− 0.23	− 0.27
I._2_	85.66	1.22	1.45	0.16	0.19	84.58	0.13	0.15
I._3_	85.85	1.37	1.63	0.61	0.72	85.03	0.49	0.58
I._4_	84.91	0.61	0.73	0.27	0.32	84.69	0.22	0.26
I._5_	84.25	0.08	0.10	0.27	0.32	84.69	0.22	0.26
	The simple correlation between predicted and realized gains = 0.680 ns							

Selection procedures	Fiber length at 2.5% span length (mm)							
	Predicted gain F_3_			Realized gain F_4_		Predicted gain F_4_		
	X_S_	G_S_	G_S_%	G_S_	G_S_%	X_S_	G_S_	G_S_%
Ped._1_	33.87	0.30	0.89	0.62	1.92	32.89	0.47	1.46
Ped._2_	33.31	− 0.18	− 0.53	0.05	0.15	32.32	0.04	0.11
Ped._3_	34.59	0.91	2.72	0.13	0.40	32.40	0.10	0.30
I._1_	33.87	0.30	0.89	0.09	0.28	32.36	0.07	0.20
I._2_	35.33	1.54	4.61	0.96	2.97	33.23	0.73	2.26
I._3_	35.34	1.56	4.65	0.66	2.05	32.93	0.50	1.55
I._4_	34.83	1.12	3.34	0.81	2.51	33.08	0.61	1.90
I._5_	34.17	0.55	1.65	0.59	1.83	32.86	0.45	1.38
	The simple correlation between predicted and realized gains = 0.702 ns							

Selection procedures	Fiber strength (PI)							
	Predicted gain F_3_			Realized gain F_4_		Predicted gain F_4_		
	X_S_	G_S_	G_S_%	G_S_	G_S_%	X_S_	G_S_	G_S_%
Ped._1_	9.50	− 0.04	− 0.39	− 0.03	− 0.27	10.88	− 0.02	− 0.19
Ped._2_	9.38	− 0.14	− 1.45	− 0.32	− 2.93	10.59	− 0.21	− 1.89
Ped._3_	9.46	− 0.07	− 0.73	− 0.26	− 2.38	10.65	− 0.17	− 1.54
I._1_	9.50	− 0.04	− 0.39	− 0.14	− 1.28	10.77	− 0.09	− 0.84
I._2_	9.58	0.03	0.27	0.44	4.03	11.35	0.28	2.55
I._3_	9.60	0.07	0.77	− 0.15	− 1.37	10.76	− 0.10	− 0.89
I._4_	9.53	− 0.01	− 0.12	− 0.05	− 0.46	10.86	− 0.03	− 0.31
I._5_	9.55	0.004	0.05	− 0.05	− 0.46	10.86	− 0.03	− 0.31
	The simple correlation between predicted and realized gains = 0.572 ns							

Selection procedures	Micronaire reading (MR)							
	Predicted gain F_3_			Realized gain F_4_		Predicted gain F_4_		
	X_S_	G_S_	G_S_%	G_S_	G_S_%	X_S_	G_S_	G_S_%
Ped._1_	4.22	0.05	1.22	− 0.02	− 0.57	4.09	− 0.03	− 0.62
Ped._2_	4.26	0.08	1.84	− 0.09	− 2.19	4.02	− 0.09	− 2.09
Ped._3_	4.23	0.06	1.37	− 0.03	− 0.81	4.08	− 0.03	− 0.84
I._1_	4.22	0.05	1.22	− 0.06	− 1.54	4.05	− 0.06	− 1.50
I._2_	4.17	0.01	0.20	0.04	0.89	4.15	0.03	0.70
I._3_	4.11	− 0.04	− 0.86	− 0.11	− 2.68	4.00	− 0.10	− 2.53
I._4_	4.20	0.03	0.75	0.00	− 0.08	4.11	− 0.01	− 0.18
I._5_	4.19	0.03	0.63	0.03	0.65	4.14	0.02	0.48
	The simple correlation between predicted and realized gains = 0.052 ns							

Ped._1_ = Direct selection for lint cotton yield/plant, Ped.2 = Direct selection for lint percentage, Ped.3 = Direct selection for lint index, I._1_ = Selection index involving yield and its components, I._2_ = Selection index involving boll weight, seeds per boll, seed index, fiber length, and Pressley index, I._3_ = Selection index involving lint percentage, lint index, fiber length and uniformity index, I._4_ = Selection index involving all characters Smith, (1936)., I._5_ = Selection index involving all characters^50^.

For PI and MR Table 4, predicted genetic response in F_3_ generation revealed desirable values by applying I._2_ for PI and MR. Also, I._2_ and I._3_ showed the desirable predicted and realized gains in F_4_ generation for PI and MR, respectively. The simple correlation between predicted and realized gains was 0.572 for PI and 0.052 for MR.

The results of this work were concluded superior five families from these indices in the F_4_ generation Table 5 where it showed that the first family was selected by five indices (Ped._1,_ Ped._2,_ Ped._3,_ I._1_ and I._5_), the second family was selected using six indices (Ped._1,_ Ped._3,_ I._1_, I._2_, I._4_ and I._5_), the third family was selected by six indices (Ped._1,_ Ped._2,_ I._1_, I._3_, I._4_ and I._5_), the fourth family was selected using five indices (Ped._1,_ I._2_, I._3_, I._4_ and I._5_ ), and the fifth family was selected using five indices (Ped._1,_ Ped._2,_ I._1_, I._2_ and I._4_). The superior five families released from these indices in the F_4_ generation Table 5 exceeded the better parent for LCY/P, B/P, BW, S/B, LI, and reasonable fiber traits. These families could be continued to further generations as breeding material for developing water deficit tolerant genotypes. Similar findings were reported by^11,12,15,31^.

**Table 5:** Means and their percent from the better parent of the superior four families scored by using two selection procedures for studied characters in the F_4_ generation.

Trait family no	Selected by	LCY/P (g)	B/P	BW (g)	S/B	SI (g)	LP%	LI (g)	UI%	FL (mm)	PI	MR
**1/2019**												
Mean	Ped._1,_ Ped._2,_ Ped._3,_ I._1_, I._5_	18.49	15.34	3.39	19.00	11.42	37.38	6.82	83.90	31.50	10.70	4.05
%		142.24	118.02	119.75	105.56	103.61	96.35	108.05	98.97	94.88	95.79	98.06
**5/2019**												
Mean	Ped._1,_ Ped._3,_ I._1_, I._2_, I._4_, I._5_	18.85	15.76	3.45	18.48	11.81	37.21	7.01	83.50	33.15	10.85	4.23
%		145.00	121.26	121.76	102.64	107.12	95.89	111.05	98.50	99.85	97.14	102.50
**8/2019**												
Mean	Ped._1,_ Ped._2,_ I._1_, I._3_, I._4_, I._5_	18.49	15.70	3.20	19.76	10.42	37.71	6.32	85.15	33.00	10.85	4.05
%		142.25	120.78	113.16	109.76	94.56	97.18	100.22	100.45	99.40	97.14	98.06
**13/2019**												
Mean	Ped._1,_ I._2_, I._3_, I._4_, I._5_	18.35	15.70	3.54	20.00	11.08	35.90	6.20	86.05	34.30	11.50	4.20
%		141.15	120.74	125.15	111.11	100.57	92.52	98.30	101.51	103.31	102.95	101.69
**15/2019**												
Mean	Ped._1,_ Ped._2,_ I._1_, I._2_, I._4_	18.29	16.00	3.30	20.35	10.08	37.84	6.13	84.20	32.50	10.50	3.90
%		140.67	123.08	116.46	113.05	91.47	97.53	97.11	99.33	97.89	94.00	94.43
**Mean selected fam**												
Mean	–	18.49	15.70	3.37	19.52	10.96	37.21	6.50	84.56	32.89	10.88	4.09
**% from F**_**4**_												
%	–	142.26	120.78	119.26	108.42	99.47	95.89	102.94	99.75	99.07	97.40	98.95
**Bp**												
–	–	13.0	13.0	2.83	18.0	11.02	38.8	6.31	84.77	33.2	11.17	4.13

Where; % = (Mean of the superior families/Mean of BP) × 100.

### Discriminant analysis (DA)

Discriminant analysis^32^ was performed using the MASS package in R software version 4.1.0^33^**.** The purpose is to identify the traits that efficiently discriminate among the three generations. These discriminant traits have been improved by the selection procedures and can be used to judge the selection efficiency. The results of linear discriminant analysis (LDA) in Table 6 cleared linear combinations of the studied traits (linear discriminants) that characterize or separate the three generations. As shown in Table 6, the first and the second discriminants (LD1 and LD2) are a linear combination of the seven traits where the percentage separations achieved by LD1 and LD2 were 99.7 and 0.3%, respectively. LI was the most crucial trait (coefficient = 5.92), followed by SI and BW (− 3.21 and 1.24) for LD1. On the other hand, LD2 was not effective in separating the three generations.

**Table 6:** Linear Discriminants of lint yield/plant (LY), boll number/plant (BN), boll weight (BW), seed number/boll (SN), seed index (SI), lint % (LP), and lint index (LI).

	LY	BN	BW	SN	SI	LP	LI	Proportion
LD1	− 0.007	− 0.001	1.240	− 0.177	− 3.206	− 0.907	5.921	0.997
LD2	− 0.504	0.689	4.282	− 0.119	0.274	0.209	− 0.339	0.003

**Figure 1:**
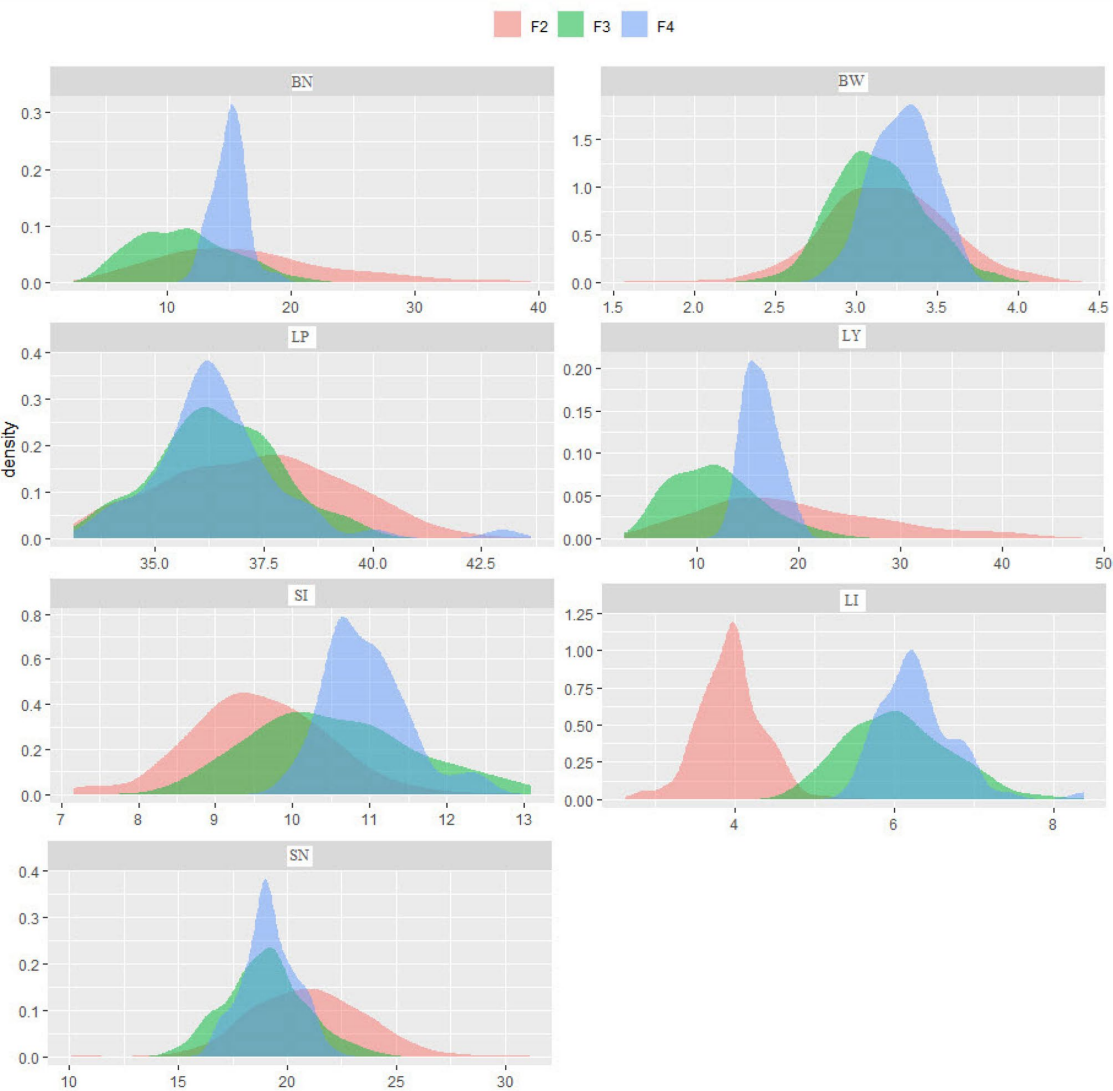
Density plot of boll number/plant (BN), boll weight (BW), lint % (LP), lint yield/plant (LY), seed index (SI), lint index (LI), and seed number/boll (SN).

Figure 1 explains the density plots of the studied traits for the three generations using smoothed kernel density function of the values. Each density plot shows both the distribution of the values and their probability. The area under the curve represents the distribution of the values of each trait, while the values on the Y-axis represent the probability of these values. The X-axis value that corresponds to the peak is the average of the feature. As shown from the figure, a higher concentration of each trait's values was demonstrated by the peaks of each density plot, which means higher probability.

In comparison, a lower concentration of the values of each trait was demonstrated by the tails of each density plot, which means lower probability. It is observed that the corresponding value to the peak of LI for F2 is different from F3 and F4, where the means of LI for F3 and F4 were higher than the mean of F2. The same trend was observed for SI, while the reverse was the case for SN. The density plots results show that the selection from F2 improved LI and SI means only and decreased the variation of all the studied traits.

**Figure 2:**
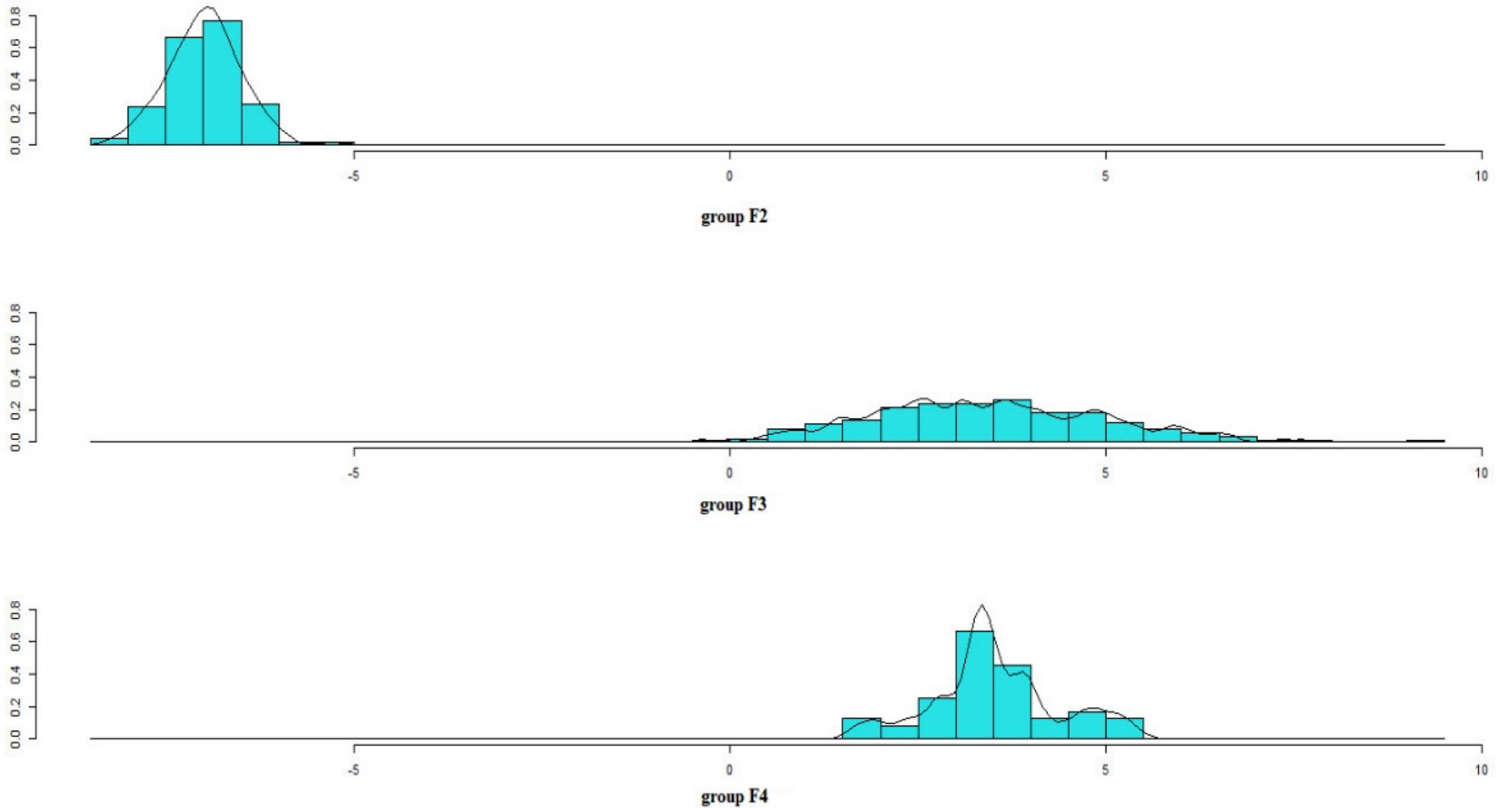
Stacked histogram for discriminant function values based on LD1.

Figure 2 shows the stacked histogram for discriminant function values based on LD1. It’s evident that no overlaps were detected between F2 and F3, and F4. However, an overlap was observed between F3 and F4. These results demonstrated that selection among F2 plants has led to an improvement in the generation F3; however, selection among F3 plants has not led to an improvement in the generation F4.

**Figure 3:**
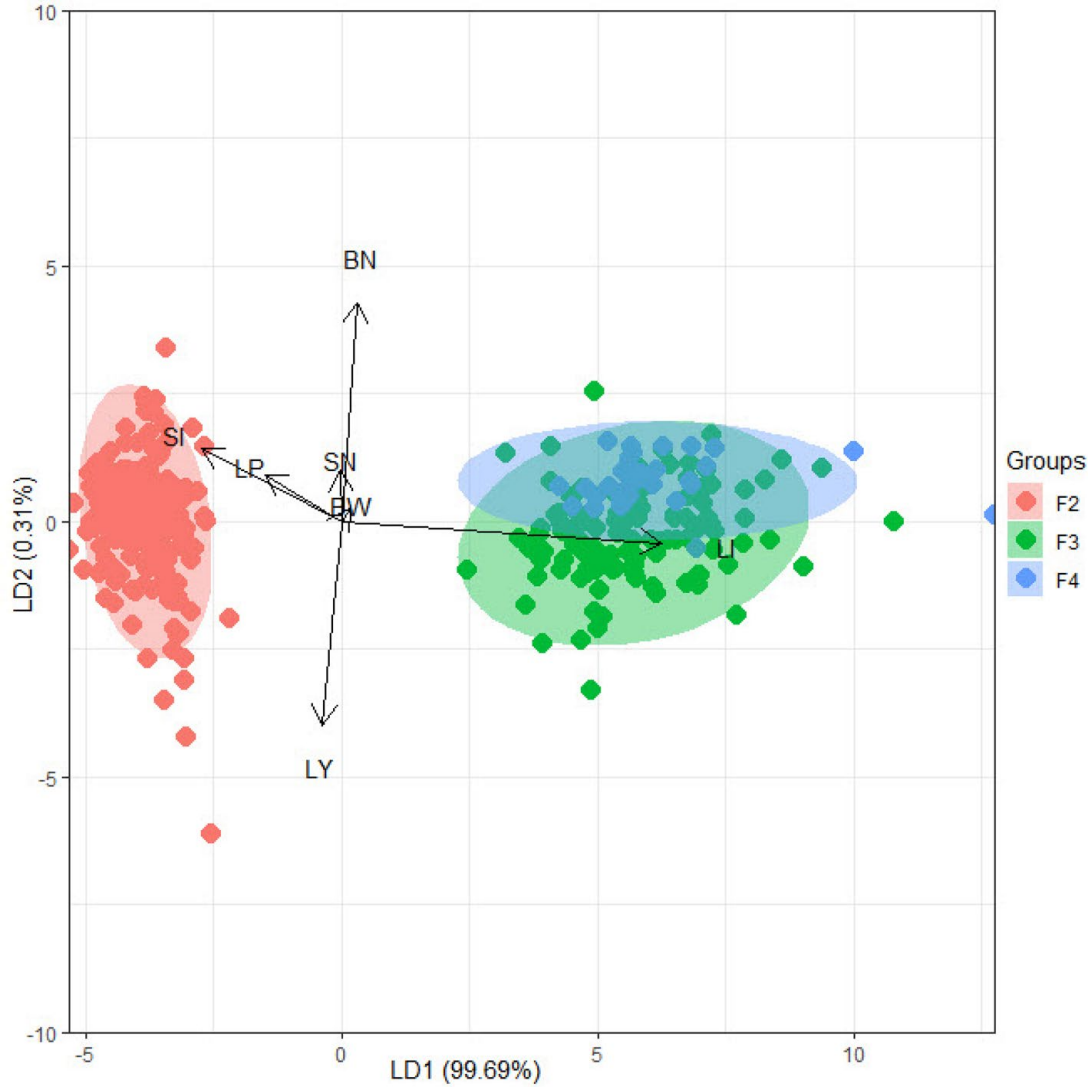
Biplot based on LD1 and LD2.

Figure 3 shows the biplot based on LD1 and LD2. It is clear that F2 was separated very clearly while an overlap was observed between F3 and F4. Based on arrows, LI explained more for F3 and F4 from F2, while SI explained more for F2 from F3 and F4 Table 7. On the other hand, BW could not explain among the three generations. At the same time, LY, BN, SN, and LP have no specific trend and can not be used to describe the variation or separate among the three generations.

**Table 7:** Means **of** lint yield/plant (LY), boll number/plant (BN), boll weight (BW), seed number/boll (SN), seed index (SI), lint % (LP), and lint index (LI) for the three generations.

Generations	LY	BN	BW	SN	SI	LP	LI
F2	19.54	16.38	3.19	21.00	9.55	37.34	3.94
F3	11.75	11.22	3.14	19.00	10.56	36.44	6.05
F4	16.25	15.10	3.29	19.20	10.95	36.29	6.23

### Path analysis

**Figure 4:**
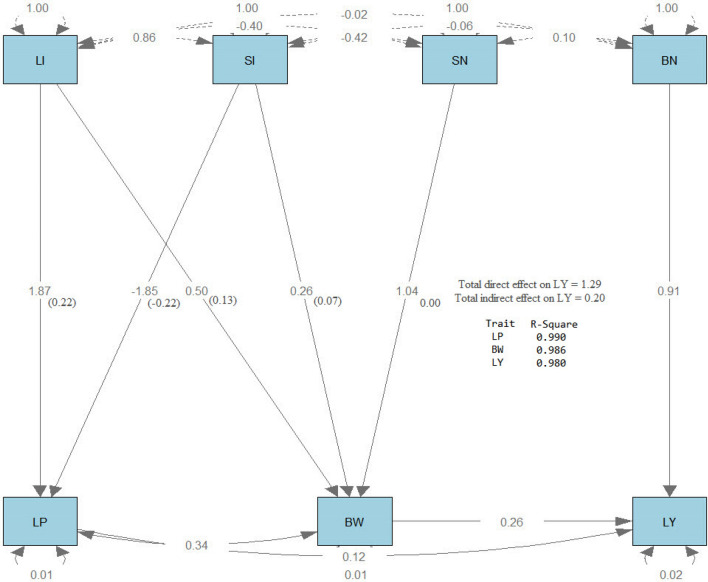
Path diagram of the direct and indirect effects of the studied traits in F2.

**Figure 5:**
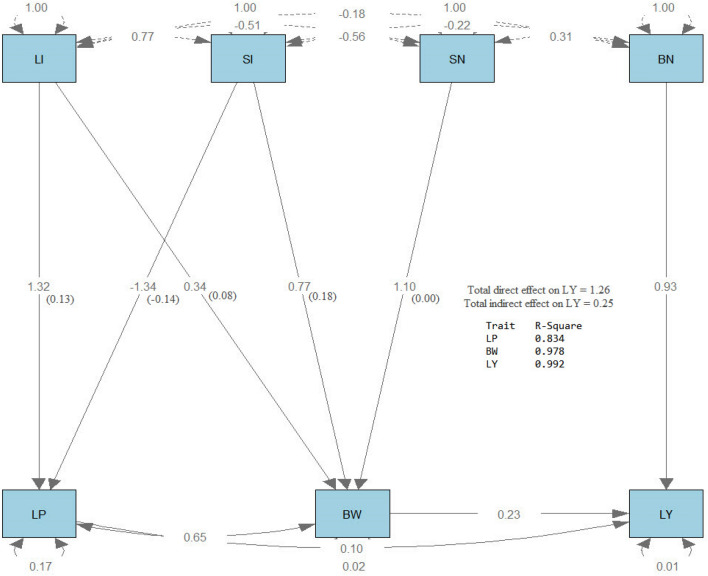
Path diagram of the direct and indirect effects of the studied traits in F3.

**Figure 6:**
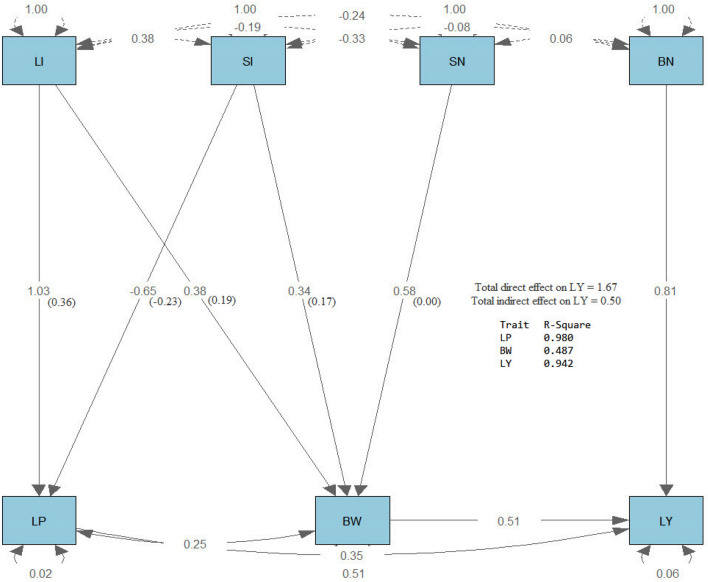
Path diagram of the direct and indirect effects of the studied traits in F4.

Path analysis^34^ was performed using the R software version 4.1.0, 2021 using the function (sem), which stans for structural equation modeling, in the (lavaan) package. Then path diagram was drawn using the process (semPaths) in the same package**.** The results of path analysis are shown on the path diagrams in Figs. 4, 5, and 6. All the path diagrams included the effects of lint index (LI) and seed index (SI) on lint % (LP), boll number/plant (BN) and seed number/boll (SN) on boll weight (BW), lint % (LP) and boll weight (BW) on lint yield/plant (LY) for the three generations. Three types of arrows characterize the path diagram. The first type is single-headed arrows (paths) used to define the causal relationships, where the trait at the tail of the needle affects the attribute at the head. The second type is double-headed arrows connecting two characteristics that define their covariance. The third type is a double-headed arrow pointing to the same trait, representing the variance of that trait. It was observed that LP was strongly affected by LI and SI, where the R^2^ values were 0.990, 0.834, and 0.980 for F2, F3, and F4, respectively. Also, BW was strongly affected by LI, SI, and SN, where the R^2^ values were 0.986, and 0.978 for F2 and F3, respectively. However, it was moderately affected in F4 (R^2^ = 0.487). LY was directly and strongly influenced by LI, SI, BN, and SN, where R^2^ values were 0.980, 0.992, and 0.942 for F2, F3, and F4, respectively. Each direct effect (standardized estimates) is shown in the middle of the arrow, and each indirect effect is shown in parenthesis beside the direct effect. The direct effect of BN on LY was more substantial than the direct effect of LP and BW in the three generations. The indirect effect of LI and SI through LP had the same trend in the three generations, where LI had a positive effect while SI had a negative effect. SN had no indirect effect on LY through BW, while LI and SI had a positive indirect effect on LY through BW in the three generations. The direct and indirect effects on LY were more significant in F4 than in F2 and F3. These more substantial effects that have been found in F4 may be due to the effect of selection, which led to a stronger relationship between Lint yield and its components and consequently stabilized the progeny of the generation.

## Discussion

The results indicated a high magnitude of genetic components and gave a possible success in the selection of the early segregating generations under water deficit stress conditions, its agree with^35–38^. The observed phenotypic and genotypic coefficients of variation were more significant in F_2_ and F_3_ than in F_4_ for all the studied traits; this was due to a reduction in genetic variability and heterozygosity as a result of using different selection procedures which exhausted a significant part of variability. Similar results agreed with those of^7,39–43^.

Mean performance in the F4 generation revealed higher than in the F3 generation for all studied characters. However, MR was lower (desirable) in F4 than F3 generation; this attributed to the possible accumulation of favorable alleles due to the efficiency of selection procedures application in this study; these results indicated the feasibility of selection for these traits. Several researchers obtained similar results by^44–48^.

Plant breeders must be concerned with the total array of economic characters and not just one character. Thus, the correlation analysis provides an excellent index to predict the corresponding change in one character at the expanse of the proportionate change in the other coefficient of phenotypic and genotypic correlations among different character combinations. Generally, genotypic correlations were higher than phenotypic correlations; this may be due to the relative stability of genotypes as the majority of them were subjected to a certain amount of selection^14,28,30^, and similar results were obtained by^11,15,16,31,49^.

Generally, from previous results under deficit water stress, direct selection for lint index (Ped._3_) was the most efficient in improving lint cotton yield/plant, bolls/plant, seeds/boll, uniformity index, and fiber length. However, the multiplicative index of^50^ involving all studied characters (I._5_) exhibited the highest values for boll weight. Also, the Ped.2 index (direct selection for lint percentage) proved to be the most efficient in improving seed and lint indexes. Direct selection for lint cotton yield/plant (Ped._1_) could produce the highest desirable values for lint percentage and micronaire reading with a relatively reasonable yield. A selection index involving yield and its components (I._2_) is recommended in improving the Pressley index.

The main objective of any breeder is to get a high yield with acceptable fiber qualities, especially under normal conditions in general^9,10,25^., and underwater deficit stress conditions in special^3,6,36,51–53^**.**This work suggested selection for favorable characters and combinations of characters under deficit water stress could be carried out at early generations because genetic variation had exhausted at early generations, and the improvement of traits could not be continued in further generations. Application of these two selection methods at the early generations of the cross-G.86 × Menoufi could improve lint yield with desirable fiber quality as one of the essential aims under deficit water stress.

## Materials and methods

### Genetic materials and selection procedures:

This work was carried out at Sakha Agricultural Research Station during the 2017, 2018, and 2019 growing seasons. The materials used were the F_2_, F_3,_ and F_4_ generations of intraspecific cotton (*Gossypium barbadense* L*.*) cross (Giza 86 × Menoufi). Giza 86 as along stable variety. It is characterized by high yield and good fiber properties. Menoufi (Giza 36) is an extra-long stable variety, the characteristics of these two parents under water deficit stress are presented in Table 8. F_2_, F_3,_ and F_4_ generations were evaluated under water deficit stress by applying one irrigation at planting and two supplemental irrigations 30 and 20 days after planting. The ordinary practices of cotton cultivation were applied.

**Table 8:** Characteristics of the cotton parental genotypes under this study.

Variety	LY/P (g)	B/P	BW (g)	S/ B	SI (g)	L %	LI (g)	UI %	FL (mm)	PI	MR
Giza 86	13.0	13.0	2.83	16.0	11.02	38.8	6.31	84.63	33.20	10.60	4.23
Menoufi (Giza36)	10.9	10.0	2.73	18.0	9.70	38.1	5.99	84.77	32.47	11.17	4.13

LY/P = Lint yield/plant, B/P = Bolls/plant, BW = Boll weight, (S/B) = Seeds/boll, SI = Seed index, L**%** = Lint percentage, LI = Lint index, FL = Fiber length, UI = Uniformity index, PI = Pressley index and MR = Micronaire reading.

In the 2017 season, F_2_ generation with original parents were grown in no replicated rows 5.0 m long with 30 cm hill space, while row to row width was kept 70 cm apart. One plant was left per hill at thinning time. Self-pollination was practiced for all F_2_ plants selfed and open-pollinated bolls/plant of 439 guarded plants. Selection of superior progenies is a procedure intensive and fiber quality trait; lint yield /plant (LY/P) (g), bolls/plant (B/P), boll weight (BW) (g), seeds/boll (S/B), seed index (SI) (g), lint percentage (LP%), lint index (LI).

Using 15% selection intensity, the plants with the highest performance for lint yield/plant, bolls/plant, boll weight, seeds/boll, seed index, lint percentage, and lint index were saved. These gave a total of 76 F_3_ selected progenies.

In the 2018 season, part of selfed seeds of 76 selected progenies was evaluated with original parents in a randomized complete blocks design with three replicates. The experimental plot consisted of a single row 5.0 m long with 30 cm hill space, while row to row width was 70 cm apart. One plant was left per hill at thinning time.

The 76 progenies were ranked using eight selection procedures. The eight superior progenies of each selection procedure were selected using 10% selection intensity. In the 2019 season, the selfed seeds of selected progenies (19) were evaluated with original parents in a randomized complete blocks design with three replicates. The experimental plot was laid out as same as carried out in 2018. Selection of superior progenies is a procedure intensive and fiber quality trait; lint yield /plant (LY/P) (g), bolls/plant (B/P), boll weight (BW) (g), seeds/boll (S/B), seed index (SI) (g), lint percentage (LP%), lint index (LI), fiber length at 2.5% span length (FL)(mm), uniformity index (UI%), Pressley index (PI), and micronaire reading (MR).

Selection procedures were as follows:

**Table:** 

Ped._1_	Direct selection for lint cotton yield/plant
Ped._2_	Direct selection for lint percentage
Ped._3_	Direct selection for lint index
I._1_	Selection index involving yield and its components
I._2_	Selection index involving boll weight, seeds per boll, seed index, fiber length, and Pressley index
I._3_	Selection index involving lint percentage, lint index, fiber length, and uniformity index
I._4_	Selection index involving all characters^24^
I._5_	Selection index involving all characters^50^

### Statistical and genetic analysis

The phenotypic (PCV) and genotypic (GCV) coefficient of variation were estimated according to^54^. The variance and covariance components from the regular randomized complete block design analysis were used to estimate the phenotypic and genotypic variances and covariances as outlined in Table 9.

**Table 9:** Analysis of variance and covariance on plot mean basis in F_3_ and F_4_ generations.

S.O.V	d.f	Mean square	Expected	
			Variance	Covariance
Replications	(r − 1)			
Families	(f − 1)	MF	σ^2^e + r σ^2^g	σe_ij_ + r σg_ij_
Error	(r − 1) (f − 1)	ME	σ^2^e	σe_ij_
Total	(rf − 1)			

Heritability in a broad sense (h^2^_b_) was calculated as follows:

$${\text{h}}_{{\text{b}}}^{2} \,({\text{in}}\,{\text{F}}_{2} \,{\text{generation}}) = \frac{{{\text{VF}}_{2} - ({\text{VP}}_{1} + {\text{VP}}_{2} )}}{{{\text{VF}}_{2} }} \times 100$$

$${\text{h}}_{{\text{b}}}^{2} \,({\text{in}}\,{\text{F}}_{3} \,{\text{and}}\,{\text{F}}_{4} \,{\text{generations}}) = \frac{{\sigma^{2} g}}{{\sigma^{2} {\text{p}}}} \times 100$$

where VF_2_ = The phenotypic variance of the F_2_ generation. VP_1_, VP_2_ = The variances of the first and second parents, respectively. σ^2^g = The genotypic variance of the F_3_ and F_4_ generations. σ^2^p = The phenotypic variances of the F_3_ and F_4_ generations. The phenotypic and genotypic correlation coefficients between studied characters in F2 and F3 and F4 generations were estimated as outlined by^55,56^**.**

The appropriate index weights (b, s) were calculated from the following formula postulated by^23,24^**:**

$$\left( {\text{b}} \right) = \left( {\text{P}} \right)^{{ - {1}}} \cdot \left( {\text{G}} \right) \cdot \left( {\text{a}} \right)$$

where (b) = Vector of relative index coefficients, (P)^−1^ = Inverse phenotypic variance-covariance matrix, (G) = Genotypic variance-covariance matrix and (a)=Vector of relative economic values on the basis of equally important, i.e., (a)_w_ = (a)_1_ = (a)_2_ = (a)_3_ = 1.

The formula suggested by^23,24^**:** Was used in calculating various selection indices:

$${\text{I}} = {\text{b}}_{{1}} {\text{x}}_{{1}} + {\text{b}}_{{2}} {\text{x}}_{{2}} + \cdots + {\text{b}}_{{\text{n}}} {\text{x}}_{{\text{n}}}$$

Predicted improvement in lint yield on the basis of an index was estimated according to the following expression:

Selection advance (SA) = SD(∑b_i_·σg_iw_)^1/2^ ^57^.

where SD denotes selection differential in standard units. bi denotes index weights for characters considered in an index. σgiw denotes genotypic covariances of the characters with yield.

Predicted genetic advance in lint yield based on direct selection was estimated from the following expression:

(ΔGw) due to selection for X_i_=K·σg_wi_/σp_i_^58^**.**

Also, the predicted response in any selected and unselected character was calculated as suggested by^57,59^.

The realized gains were calculated as deviation of generation mean for each character from the procedure mean of that character.

## Conclusion

In this study, the result under deficit water stress**,** direct selection for lint index (Ped._3_) was the most efficient in improving lint cotton yield/plant, bolls/plant**,** seeds/boll, uniformity index, and fiber length**.** However, the multiplicative index involving all studied characters (I._5_) exhibited the highest values for boll weight. Also, the Ped.2 index (direct selection for lint percentage) proved to be the most efficient in improving seed and lint indexes. Direct selection for lint cotton yield/plant (Ped._1_) could produce the highest desirable values for lint percentage and micronaire reading with a relatively reasonable yield. A selection index involving yield and its components (I._2_) is recommended for improving the Pressley index**.**

## Abbreviations

LY/P: Lint yield/plant
B/P: Bolls/plant
BW: Boll weight
(S/B): Seeds / boll
SI: Seed index
LP%: Lint percentage
LI: Lint index
FL: Fiber length
UI: Uniformity index
PI: Pressley index
MR: Micronaire reading
